# Addition of dairy lipids and probiotic *Lactobacillus fermentum* in infant formula programs gut microbiota and entero-insular axis in adult minipigs

**DOI:** 10.1038/s41598-018-29971-w

**Published:** 2018-08-03

**Authors:** Marion Lemaire, Samir Dou, Armelle Cahu, Michèle Formal, Laurence Le Normand, Véronique Romé, Isabelle Nogret, Stéphanie Ferret-Bernard, Moez Rhimi, Isabelle Cuinet, Cécile Canlet, Marie Tremblay-Franco, Pascale Le Ruyet, Charlotte Baudry, Philippe Gérard, Isabelle Le Huërou-Luron, Sophie Blat

**Affiliations:** 1 INRA, INSERM, Univ Rennes, Nutrition Metabolisms and Cancer, NuMeCan, Rennes, France; 2 Lactalis R&D, Retiers, France; 3 PEGASE, INRA, Agrocampus Ouest, Saint-Gilles, France; 4 Micalis, INRA, AgroParisTech, Univ Paris-Saclay, Jouy-en-Josas, France; 5 Toxalim, INRA, Univ Toulouse, ENVT, INP-Purpan, UPS, PF MetaToul-AXIOM, Toulouse, France

## Abstract

Clinical and animal studies have demonstrated beneficial effects of early consumption of dairy lipids and a probiotic, *Lactobacillus fermentum* (Lf), on infant gut physiology. The objective of this study was to investigate their long-term effects on gut microbiota and host entero-insular axis and metabolism. Piglets were suckled with a milk formula containing only plant lipids (PL), a half-half mixture of plant lipids and dairy lipids (DL), or this mixture supplemented with Lf (DL + Lf). They were weaned on a standard diet and challenged with a high-energy diet until postnatal day 140. DL and DL + Lf modulated gut microbiota composition and metabolism, increasing abundance of several *Clostridia* genera. Moreover, DL + Lf specifically decreased the faecal content of 2-oxoglutarate and lysine compared to PL and 5-aminovalerate compared to PL and DL. It also increased short-chain fatty acid concentrations like propionate compared to DL. Furthermore, DL + Lf had a beneficial effect on the endocrine function, enhancing caecal GLP-1 and GLP-1 meal-stimulated secretion. Correlations highlighted the consistent relationship between microbiota and gut physiology. Together, our results evidence a beneficial programming effect of DL + Lf in infant formula composition on faecal microbiota and entero-insular axis function.

## Introduction

According to the developmental origins of health and disease (DOHaD), the early life environment may have long-lasting effects on health^1–3^. Underlying mechanisms to such a programming remain largely unknown but gut microbiota has recently been identified as a potential key actor, able to induce long-lasting changes in host intestinal functions^4^. Gut microbiota dysbiosis has therefore been associated with an increased susceptibility to metabolic disorders^5^.

The early postnatal period is a crucial window for the interplay between diet, gut microbiota and host metabolism^6–8^. Neonatal nutrition may modulate early implantation of gut microbiota and therefore exert long-lasting metabolic and physiological effects in adulthood^9^. Differences in gut microbiota diversity and composition have been demonstrated from the first week of life between breastfed and formula-fed infants^10^, the last ones being at higher risk for later insulin resistance and type 2 diabetes^11,12^. The structure and composition of the lipid matrix and microbiota of breast milk could partly explain its long-term protective effects.

Indeed, human milk contains high concentrations of medium-chain fatty acids associated with a complex structure, named milk fat globules^13,14^. In contrast, the lipid matrix of most formulas on the market is exclusively made of vegetable oils containing mostly long-chain fatty acids^13^. We demonstrated that the addition of dairy lipids in formulas had short-term benefits on digestion, gut physiology and microbiota in a piglet model^15^. Moreover, mimicking the milk fat globule size and coating was associated with long-term beneficial effects in a murine model, with a decreased fat accumulation and improved metabolic profile^16^.

Besides, recent data emphasize that breast milk has a diversified bacterial ecosystem with over 200 different genera and 700 species^17–19^. These bacteria would account for almost one-third of total bacteria present in the gut of breastfed infants during the first month of life^20^. The addition of probiotics in infant formulas may, therefore, be of great interest. Among probiotics, *Lactobacillus fermentum* CECT 5716 (Lf), a lactic acid bacteria originally isolated from human milk^21^, has been proven to exert beneficial short-term effects, in particular on immediate gut microbiota composition^22^, prevention of infections^23^ and barrier dysfunction^24^.

If short-term benefits of dairy lipids and Lf supplied separately have previously been demonstrated on gut physiology and microbiota composition, their long-term outcomes on gut microbiota and glucose metabolism remain unknown. Moreover, the effects of dairy lipids and Lf supplied together have not been evaluated yet. We hypothesized that dairy lipids and Lf effects on gut microbiota and physiology in the neonate could have long-term consequences in adulthood on gut microbiota, host entero-insular axis and metabolism. We used a diet-induced overweight Yucatan minipig model^25^ to reveal a potential latent programming effect induced by the infant formula composition.

## Results

**Figure 1:**
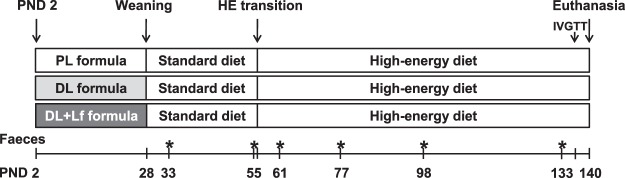
Study design from PND2 to PND140. Piglets were allowed to suckle the dam until PND2. They were then separated from their dam and fed with an automatic formula feeder until weaning, at PND 28. Formulas contained as lipids either: only plant lipids (PL), a half-half mixture of plant and dairy lipids (DL) or a half-half mixture of plant and dairy lipids supplemented with Lf (DL + Lf). From PND28 to 56, pigs were fed commercial starter diets and then challenged with a HE diet until euthanasia, at PND140. Glucose homeostasis was evaluated at PND133 via an IVGTT and fasting plasma was used for metabolomics analysis. Faeces (*) were collected at PND33 (after weaning), PND55 (before HE transition), PND61 (after HE transition), PND77, PND98 and PND135, 3, 6 and 12 weeks after HE transition, respectively. At euthanasia, faecal content was collected for metabolomics and microbiota analysis and the entero-insular axis was investigated. PND, postnatal day; HE, high-energy; IVGTT, intravenous glucose tolerance test.

The study design and the composition of the infant formulas used in this study are described in Fig. 1 and Table 1 (see Methods for details).

**Table 1:** Composition of infant formulas.

g/100 g of powder	PL	DL	DL + Lf
Proteins	17.8	17.9	17.9
Lipids	43.6	44.7	44.6
Carbohydrates	33.1	32.3	32.2
Minerals	3.5	3.4	3.4
Energy (kJ)	2476	2506	2501
*Lactobacillus fermentum* CECT 5716 (Lf) (CFU/100 g of powder)	—	—	1.9E + 08

Formulas contained as lipids either: only plant lipids (PL), a half-half mixture of plant and dairy lipids (DL) or a half-half mixture of plant and dairy lipids supplemented with Lf (DL + Lf). The three infant formulas were produced in one batch of production each and used for the three replications of the experiment. The composition reading is that of this one batch production. Formulas were rehydrated at 20% of dry extract. Lipid sources of the PL formula were palm oil (71.7%), rapeseed oil (23.2%) and refined sunflower oil (5.1%); those of the DL and DL + Lf formulas were cream (53.4%), rapeseed oil (21.1%), refined sunflower oil (13.1%) and high oleic sunflower oil (12.4%). CFU, colony*-*forming unit.

### Effects of dairy lipids and Lf on adult gut microbiota

#### Diversity

**Figure 2:**
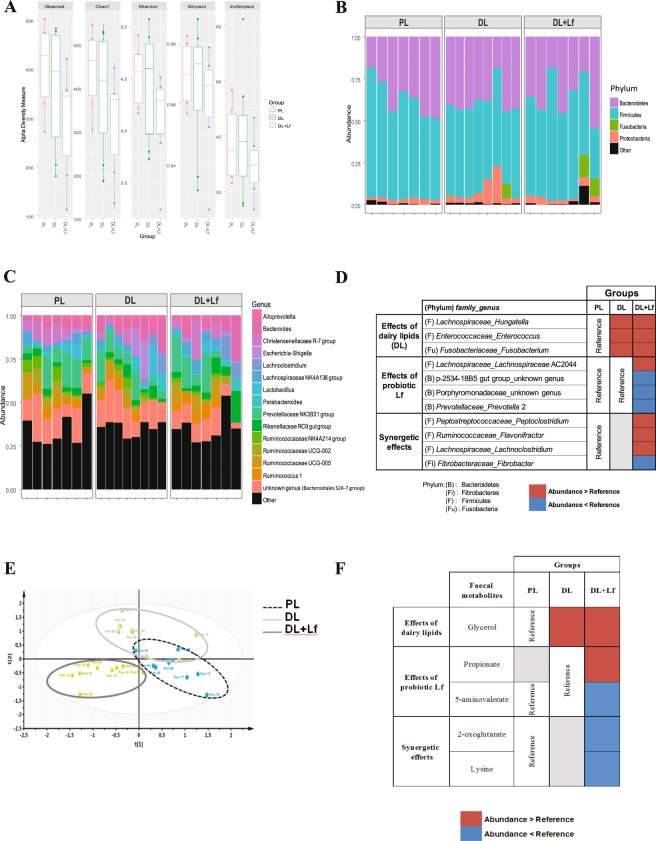
Composition and metabolism of gut microbiota. (**A**) α diversity indices in faecal samples of adult PL, DL and DL + Lf pigs. (**B**) Bacteria composition at phylum and (**C**) genus levels (relative abundance) in faecal samples of adult PL, DL and DL + Lf pigs. (**D**) Discriminating genera in the faeces of adult PL, DL and DL + Lf pigs. (**E**) Two-dimensional PLS-DA score plot of integrated ^1^H-NMR spectra of adult PL, DL and DL + Lf faecal samples. Each dot represents an animal, projected onto first (horizontal axis) and second (vertical axis) PLS-DA variables. The ellipse determines the 95% confidence interval, which is drawn using Hotelling’s T^2^ statistic. PLS-DA model parameters: four latent variables included, R^2^Y (cum) = 90.4% (% of variance explained by the model), Q^2^ = 0.66 (predictive ability of the model) and p-value = 0.02. (**F**) Discriminating faecal metabolites between adult PL, DL and DL + Lf pigs. Formulas contained as lipids either: only plant lipids (PL, n = 6–9), a half-half mixture of plant and dairy lipids (DL, n = 7–8) or a half-half mixture of plant and dairy lipids supplemented with Lf (DL + Lf, n = 7–9).

Alpha diversity (species richness, Chao1, Simpson, Shannon and inverse Simpson indices) measured on postnatal day (PND) 140 faecal samples was not affected by the composition of the infant formula (Fig. 2A and Supplementary Table 1), nor was beta diversity (weighted and unweighted Unifrac, Jaccard and Bray-Curtis distances) (Supplementary Table 1). To evaluate the contribution of diet to the pig microbiota, a permutational multivariate analysis of variance was applied using the distance matrices. The infant formula composition accounted for 9.8 to 12.1% of the total variation between samples.

#### Composition

Eight phyla, 50 families and 171 genera were identified in faecal contents. The relative abundances of predominant bacterial phyla and genera are shown in Fig. 2B,C. Firmicutes constituted the dominant phylum in all groups, accounting for an average of 55% of the total sequences (Fig. 2B). Other bacterial phyla were Bacteroidetes (37%), Proteobacteria (4.6%), Fusobacteria (1.8%), Actinobacteria (1.0%), Tenericutes (0.30%), Fibrobacteres (0.12%), and Deferribacteres (0.08%). Genus-level analysis showed that the top six genera were *Prevotellaceae* NK3B31 group, unknown genus (*Bacteroidales* S24-7 group), *Bacteroides*, *Christensenellaceae* R-7 group, *Rikenellaceae* RC9 gut group and *Ruminococcaceae* UCG-005 (Fig. 2C).

482 operational taxonomic units (OTUs) allocated between seven phyla, 36 families and 123 genera contributed to at least 0.1% of the microbial community in at least 5% of the samples. The fat matrix and the addition of the probiotic Lf had multiple effects (a fat matrix effect, PL *vs*. DL and PL *vs*. (DL and DL + Lf); a probiotic effect, DL *vs*. DL + Lf and (DL and PL) *vs*. DL + Lf; and a synergetic effect, PL *vs*. DL + Lf) on the composition of the faecal microbiota (Fig. 2D).

Among the 78 discriminating OTUs, 25 (32%) were specific to the lipid matrix whereas 26 (33%) were specific to the probiotic Lf. OTUs within the Firmicutes phylum were most affected by the formula composition (67% of the discriminating OTUs) (Supplementary Table 1). At phylum level, the addition of dairy lipids was associated with increased abundance of Fusobacteria and decreased abundances of Firmicutes and Tenericutes. Probiotic Lf did not have any impact on phyla abundances. At the genus level, 11 genera were affected by the formula composition, among which five belonged to *Clostridiales* order. The addition of dairy lipids was associated with higher abundances of *Hungatella*, *Enterococcus* and *Fusobacterium* (DL and DL + Lf *vs*. PL) (Fig. 2D). The addition of Lf stimulated a significant expansion of *Lachnospiraceae* AC2044 group and reduction of *Prevotella* 2 and two unknown genera belonging to p-2534-18B5 gut group and *Porphyromonadaceae* families (DL + Lf *vs*. PL and DL). Dairy lipids and probiotic Lf addition had synergetic effects on four genera, increasing three genera belonging to *Clostridiales* order (*Peptoclostridium*, *Flavonifractor* and *Lachnoclostridium)*, and decreasing *Fibrobacter*.

#### Metabolic activity

Short-chain fatty acid (SCFA) concentration was measured at different time points on faecal samples and at euthanasia on caecal and colon contents. Total faecal SCFA production did not differ with age whatever the formula (p = 0.15) and was similar between groups from weaning until PND77 (Table 2). At PND98, six weeks after the beginning of the high-energy (HE) diet, total faecal SCFAs, acetate and valerate concentrations tended (p = 0.09, 0.07 and 0.07, respectively) to increase and isobutyrate concentration was significantly increased (p = 0.02) in DL + Lf compared to DL. Besides, isovalerate was significantly (p = 0.03) increased in both PL and DL + Lf compared to DL. At PND133, faecal propionate level tended (p = 0.08) to increase in DL + Lf compared to DL. No differences were observed at euthanasia for total SCFAs in caecal and colonic contents (Table 2).

**Table 2:** Short-chain fatty acid concentrations in faeces and digesta of adult PL, DL and DL + Lf pigs.

Faecal SCFAs (mmol/kg)	PL	DL	DL + Lf	p-value
				Diet effect
Total SCFAs PND33	143 ± 21	117 ± 15	155 ± 24	0.34
Total SCFAs PND55	113 ± 8	116 ± 10	115 ± 15	0.99
Total SCFAs PND61	126 ± 9	111 ± 16	122 ± 14	0.74
Total SCFAs PND77	125 ± 17	129 ± 15	120 ± 12	0.92
Total SCFAs PND98*	133 ± 13^a,b^	102 ± 8^a^	139 ± 18^b^	**0.09**
Acetate*	77.0 ± 7.0^a,b^	60.4 ± 4.4^a^	77.6 ± 8.6^b^	**0.07**
Propionate	24.8 ± 2.7	20.1 ± 1.6	24.3 ± 3.3	0.37
Butyrate	16.9 ± 2.7	12.1 ± 1.4	20.5 ± 4.5	0.26
Isobutyrate	3.99 ± 0.52^a,b^	2.48 ± 0.33^a^	5.03 ± 0.93^b^	**0.02**
Valerate*	3.25 ± 0.35^a,b^	2.36 ± 0.24^a^	3.81 ± 0.61^b^	**0.07**
Isovalerate*	6.55 ± 0.97^b^	3.58 ± 0.61^a^	7.23 ± 1.30^b^	**0.03**
Total SCFAs PND133	116 ± 18	77.7 ± 10.7	120 ± 12	0.12
Acetate	68.2 ± 9.8	48.9 ± 5.4	73.1 ± 7.8	0.13
Propionate*	21.7 ± 3.9^a,b^	13.7 ± 2.7^a^	21.7 ± 1.9^b^	**0.08**
Butyrate	15. 6 ± 3.7	7.57 ± 1.95	14.1 ± 2.1	0.15
Isobutyrate	2.84 ± 0.47	2.09 ± 0.47	2.95 ± 0.32	0.22
Valerate	2.70 ± 0.41	1.79 ± 0.22	2.83 ± 0.33	0.11
Isovalerate	4.27 ± 0.60	3.35 ± 0.42	5.16 ± 0.62	0.12
**Relative organ weight (PND140) (g/kg of body weight)**	**PL**	**DL**	**DL + Lf**	**Diet effect**
Caecum full	2.97 ± 0.21	3.90 ± 0.33	3.13 ± 0.47	0.17
Caecum empty	1.50 ± 0.07	1.63 ± 0.12	1.54 ± 0.15	0.75
Colon full	27.6 ± 0.9	26.9 ± 1.0	28.6 ± 1.7	0.63
Colon empty	18.6 ± 0.4	19.4 ± 0.7	19.7 ± 0.8	0.37
**Total SCFAs (mmol/kg)**	**PL**	**DL**	**DL + Lf**	**Diet effect**
Caecum	78.8 ± 9.2	97.2 ± 4.3	80.9 ± 15.5	0.46
Colon	67.1 ± 6.9	66.9 ± 6.0	68.6 ± 9.9	0.99

Formulas contained as lipids either: only plant lipids (PL, n = 9), a half-half mixture of plant and dairy lipids (DL, n = 7–8) or a half-half mixture of plant and dairy lipids supplemented with Lf (DL + Lf, n = 8–9). SCFA, short-chain fatty acid; HE, high-energy diet; PND, postnatal day. ^a,b^Labelled means in a row without a common letter differ significantly (p < 0.05).

*Total SCFAs PND98: significant diet × sex (DL + Lf females > DL + Lf and DL males) and sex (females > males) effects; acetate PND98: significant diet × sex (DL + LF females > DL and DL + Lf males) and sex (females > males) effects; isovalerate and valerate: significant sex effet (females > males); propionate PND133: significant sex effect (males > females). Data are expressed as the mean ± SEM.

High-resolution ^1^H-NMR spectra were recorded from faecal contents at PND140. A valid and robust PLS-DA model was constructed (Fig. 2E). The DL + Lf group was clearly separated from the two other groups which shared an overlapping area. Among the 40 identified metabolites, five were statistically different between groups (Fig. 2F). Glycerol was increased in DL and DL + Lf compared to PL, and 2-oxoglutarate and L-lysine were decreased in DL + Lf compared to PL. 5-aminovalerate was decreased in DL + Lf compared to PL and DL whereas propionate was increased in DL + Lf compared to DL.

The correlations between the microbial composition and metabolites were analysed using Spearman correlations (Supplementary Table 2). Several discriminating genera were correlated to each other. For example, *Lachnoclostridium* was positively associated with *Flavonifractor* (R = 0.77, p < 0.01) and *Hungatella* (R = 0.57, p = 0.01) and negatively associated with *Lachnospiraceae* AC2044 group (R = −0.61, p < 0.01). Several discriminating genera were also correlated to discriminating metabolites or SCFA concentrations. For instance, *Hungatella* was positively associated with caecal acetate (R = 0.52, p = 0.01) whereas *Prevotella* 2 was negatively associated with faecal propionate (R = −0.54, p = 0.01) and *Peptoclostridium* was negatively associated with 5-aminovalerate (R = −0.51, p = 0.01).

### Effects of dairy lipids and Lf on adult GLP-1 secretion

**Figure 3:**
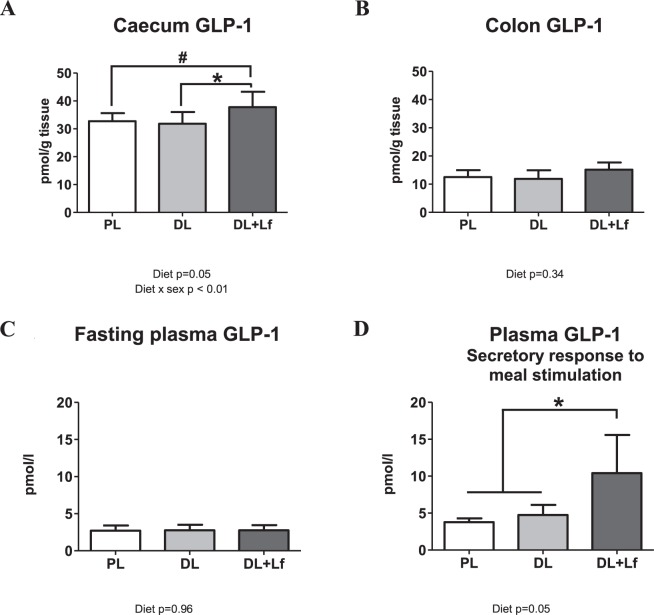
Intestinal endocrine function. (**A**) Caecal and (**B**) colon GLP-1 of adult PL, DL and DL + Lf pigs. (**C**) Fasting plasma GLP-1 and (**D**) plasma GLP-1 secretory response to meal stimulation of adult PL, DL and DL + Lf pigs. Formulas contained as lipids either: only plant lipids (PL, n = 7–9), a half-half mixture of plant and dairy lipids (DL, n = 6–8) or a half-half mixture of plant and dairy lipids supplemented with Lf (DL + Lf, n = 4–9). Data are shown as Means ± SEM. GLP-1, Glucagon-like peptide-1. A significant diet × sex effect was observed for caecal GLP-1, with the DL + Lf females having a higher concentration than DL and PL females.

Enteroendocrine and glucagon-like peptide-1 (GLP-1)-secreting cell densities, as well as the percentage of GLP-1-secreting cells amongst all enteroendocrine cells were similar between groups in the caecum and the colon (Supplementary Table 3). However, caecal GLP-1, but not colon GLP-1, was increased in DL + Lf pigs compared to PL and DL groups (Fig. 3A,B). In addition, plasma GLP-1 secretory response to meal stimulation was increased in DL + Lf group compared to PL and DL groups (Fig. 3D), although fasting plasma GLP-1 was not different between groups (Fig. 3C). Even though no significant difference was observed between groups for the endocrine pancreas anatomy and its insulin content (Supplementary Table 3), caecal GLP-1 secreting L-cell density was positively correlated to caecal GLP-1, to plasma GLP-1 secretory response to meal stimulation, and to the endocrine pancreas (% endocrine tissue) (Supplementary Table 4). Similarly, colon GLP-1 secreting L-cell density was positively correlated to GLP-1 concentration in the colon and fasting plasma GLP-1 (Supplementary Table 4).

**Figure 4:**
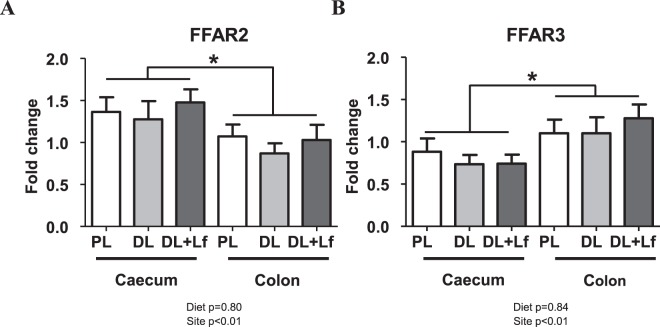
Relative caecal and colonic expression of *FFAR2* (**A**) and *FFAR3* (**B**) in adult PL, DL and DL + Lf pigs. Formulas contained as lipids either: only plant lipids (PL, n = 9), a half-half mixture of plant and dairy lipids (DL, n = 7) or a half-half mixture of plant and dairy lipids supplemented with Lf (DL + Lf, n = 8). Data represent the *ratio* of the relative expression of groups to colon PL group (±SEM). *FFAR*, Free Fatty Acid Receptor.

Independently of formula composition, free fatty acid receptor (*FFAR*) *2* expression was significantly increased in the caecum compared to the colon (Fig. 4A) whereas *FFAR3* expression was significantly increased in the colon compared to the caecum (Fig. 4B). *FFAR3* expressions in both the caecum and the colon were positively correlated to the percentage of GLP-1-secreting cells amongst enteroendocrine cells (Supplementary Table 4).

**Figure 5:**
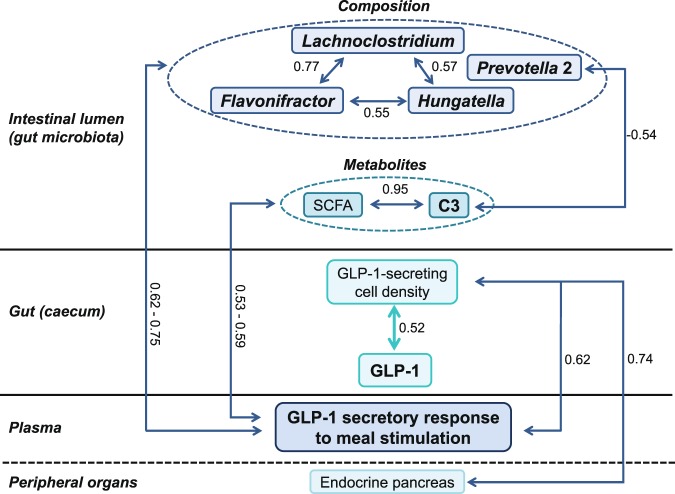
Significant correlations between gut microbiota composition, luminal metabolites and host entero-insular axis parameters. Within the gut microbiota, the three genera *Flavonifractor*, *Lachnoclostridium* and *Hungatella* were positively correlated. They all belong to the *Clostridia* class, including SCFA-producing bacteria. These genera were also positively correlated to plasma GLP-1 secretory response to meal stimulation. Plasma GLP-1 secretory response to meal stimulation was, as expected, positively associated with luminal SCFA concentrations, specifically propionate. The percentage of caecal GLP-1 secreting-cells amongst enteroendocrine cells was positively correlated to the caecal GLP-1 content, to the plasma GLP-1 secretory response to meal stimulation and to the pancreatic endocrine tissue, in accordance with the well-known trophic effect of GLP-1 on β cells. These results highlight potential mechanisms between bacteria composition and metabolism, and host metabolism. GLP-1, Glucagon-like peptide-1; SCFA, short-chain fatty acid; C3, propionate. R > |0.50| and p < 0.05. In bold: significant effect of the infant formula composition.

To investigate the role of the gut microbiota as an actor of nutritional programming, its composition and metabolic activity were correlated to the entero-insular axis-related parameters (Supplementary Table 5 and Fig. 5). Faecal SCFA concentrations measured after six weeks of HE diet were positively correlated to caecal GLP-1 secreting L-cell density (total SCFAs, acetate, isobutyrate), caecal GLP-1 concentration (propionate) and plasma GLP-1 secretory response to meal stimulation (total SCFAs, acetate, butyrate). Plasma GLP-1 secretory response to meal stimulation was also positively correlated to several SCFAs concentrations measured at PND140 (total SCFAs, acetate, propionate, butyrate, isobutyrate, valerate and isovalerate, colon butyrate) and to three discriminating genera (*Flavonifractor*, *Hungatella* and *Lachnoclostridium*). Caecal GLP-1 concentration was positively correlated to butyrate concentration (faeces at PND77 and colon at PND140) (Supplementary Table 5). *Lachnospiraceae* AC2044 group was positively correlated to colon GLP-1 secreting L-cell density and fasting plasma GLP-1 (Supplementary Table 5).

### Effects of dairy lipids and Lf on adult metabolic adaptation to a three-months HE diet challenge

#### Growth, food intake and body composition

Even if formula composition had an impact on weight gain during the suckling period, no significant difference in growth and food intake was observed between the three groups after weaning and throughout the experiment (Supplementary Fig. 1). Three months of HE diet induced a substantial increase in backfat (reflect of adiposity), regardless of the formula received in early life and all groups had similar body and relative organ weights at euthanasia (PND140) (Supplementary Table 6).

#### Inflammation and metabolic disorders

Three months of HE diet led to a weak inflammation as indicated by a plasma level of haptoglobin slightly above 2.5 g/L. However, the composition of infant formulas received in early life had no significant long-term impact on plasma haptoglobin and TNFα levels, and on caecal and colon alkaline phosphatase activity (Supplementary Table 7). Only plasma IL-1β was higher in DL + Lf compared to PL pigs (Supplementary Table 7). Cytokine secretions (IFNγ, TNFα, IL-10) of peripheral blood mononuclear cells (PBMCs) stimulated with concanavalin A (ConA) mitogen or lipopolysaccharide (LPS) were not significantly different between groups (Supplementary Table 7). Finally, plasma IL-1β was negatively correlated to an unknown genus belonging to the *Porphyromonadaceae* family (R = −0.53, p = 0.02).

The composition of infant formulas had no significant long-term impact on lipid profile and plasma fasting glucose and insulin concentrations at PND133 (Table 3). Besides, the intravenous glucose tolerance test (IVGTT), aiming at challenging glucose homeostasis, did not reveal significant differences between groups in glucose and insulin profiles as well as in calculated indexes (Table 3 and Supplementary Fig. 2). Even though infant formulas had no significant impact on metabolism and liver sensitivity to insulin with regards to genes related to insulin action (*INSR* and *GLUT*2) and *PEPCK*, the expression of liver glucose-6-phosphatase (G6Pase) was decreased in DL and DL + Lf groups compared to PL (Table 4). In addition, fasting insulin was positively correlated to *Lachnospiraceae* AC2044 group (R = 0.53, p = 0.02) (Supplementary Table 5). Plasma GLP-1 secretory response to meal stimulation was negatively correlated to *INSR* expression in the liver (R = −0.66, p = 0.01) (Supplementary Table 4), itself negatively correlated to *Flavonifractor* (R = −0.52, p = 0.02) (Supplementary Table 5).

**Table 3:** Effects of infant formula composition on plasma lipid profile and metabolic outcomes of the intravenous glucose tolerance test (IVGTT) of adult PL, DL and DL + Lf pigs.

Lipid profile (mmol/l)	PL	DL	DL + Lf	p-value
				Diet effect
NEFA	0.63 ± 0.03	0.66 ± 0.05	0.58 ± 0.07	0.61
Cholesterol*	2.99 ± 0.17	2.89 ± 0.14	2.92 ± 0.14	0.36
HDL-cholesterol	1.24 ± 0.08	1.23 ± 0.12	1.12 ± 0.11	0.55
Triglycerides	0.28 ± 0.03	0.29 ± 0.03	0.25 ± 0.04	0.70
**Fasting glucose metabolism**				
Glucose (mmol/l)	5.43 ± 0.10	5.16 ± 0.15	5.48 ± 0.15	0.24
Insulin (µIU/ml)	28.3 ± 3.2	32.9 ± 3.1	29.2 ± 3.1	0.38
HOMA-IR	6.83 ± 0.77	7.63 ± 0.88	7.14 ± 0.79	0.63
**Metabolic outcomes**				
Incremental glucose AUC (iAUCG, mmol/l)	130 ± 5	148 ± 10	138 ± 10	0.23
Incremental insulin AUC* (iAUCI, µIU/ml)	3339 ± 234	3221 ± 256	3384 ± 435	0.43
AIR _0–6min_ (µIU/ml)	145 ± 10	129 ± 8	139 ± 10	0.45
K_G_* (% glucose disappearance/min)	4.81 ± 0.24	4.56 ± 0.34	4.66 ± 0.37	0.56
Incremental insulin AUC/				
Incremental glucose AUC*	25.7 ± 1.5	22.1 ± 1.8	24.3 ± 2.4	0.17
S_2_* (min^−1^.(µIU. ml^−1^)^−1^)	4.85 ± 0.35	4.46 ± 0.62	5.08 ± 1.00	0.44
S_I_* (10^–4^.min^−1^.(µIU.ml^−1^)^−1^)	5.01 ± 0.51	5.39 ± 1.17	6.00 ± 1.45	0.15
S_G_ (10^−2^.min^−1^)	3.22 ± 0.46	2.82 ± 0.09	3.14 ± 0.38	0.90

Formulas contained as lipids either: only plant lipids (PL, n = 9), a half-half mixture of plant and dairy lipids (DL, n = 6–8) or a half-half mixture of plant and dairy lipids supplemented with Lf (DL + Lf, n = 6–7). NEFA, Non-Esterified Fatty Acids; HDL-cholesterol, High-Density Lipoprotein cholesterol; LDL, Low-density Lipoproteins; HOMA-IR, homeostasis model assessment of insulin resistance; AUC, area under the curve; AIR, acute insulin response; K_G_, rate of glucose disappearance; S_2_ and S_I_, insulin sensitivity indexes; S_G_, glucose efficiency index. Data are expressed as the mean ± SEM.

*A sexual dimorphism was observed: cholesterol level (p < 0.01) was higher in females than in males, AUC and Incremental insulin AUC/incremental glucose AUC were increased and K_G_, S_2_ and S_I_ decreased in in males compared to females (p < 0.05).

**Table 4:** Relative expression of genes related to insulin sensitivity in liver of adult PL, DL and DL + Lf pigs.

Liver	PL (n = 8)	DL (n = 8)	DL + Lf (n = 8)	p-value
				Diet effect
*G6Pase*	1.08 ± 0.15^b^	0.87 ± 0.12^a^	0.82 ± 0.08^a^	**0.04**
*GLUT2*	1.02 ± 0.08	0.92 ± 0.07	0.88 ± 0.07	0.36
INSR*	1.01 ± 0.06	1.01 ± 0.04	0.93 ± 0.03	0.13
PEPCK*	1.08 ± 0.16	0.87 ± 0.12	0.91 ± 0.09	0.42

Values are expressed as the *ratio* of the relative expression of DL and DL + Lf groups to PL group (±SEM).

Formulas contained as lipids either: only plant lipids (PL), a half-half mixture of plant and dairy lipids (DL) or a half-half mixture of plant and dairy lipids supplemented with Lf (DL + Lf). *G6Pase*, Glucose-6-Phosphatase; *GLUT2*, Glucose transporter 2; *INSR*, Insulin Receptor; *PEPCK*, PhosphoEnolPyruvate CarboxyKinase. ^a,b^Labelled means in a row without a common letter differ significantly (p < 0.05).

*INSR and *PEPCK* expressions were increased (p < 0.05) in females compared to males.

## Discussion

The objective of this study was to investigate the potential long-term programming effect of early infant formula consumption containing dairy lipids with or without probiotic Lf on gut microbiota and host entero-insular axis and metabolism. Our hypothesis was that early consumption of dairy lipids and Lf may program gut microbiota composition and metabolism and therefore affect the long-term response of adult pigs facing a HE diet. The HE diet was administered to highlight a potential latent programming effect of the formula composition. Our results demonstrated that both the addition of dairy lipids and probiotic Lf in infant formulas had an impact on gut microbiota composition. However, only the addition of Lf had a noticeable long-term promoting effect on SCFA production (especially propionate) and GLP-1 secreting function.

The first and main result of this study is that the composition of the infant formula fed in early life had a long-term programming effect on gut microbiota composition (from phylum to genus) and metabolic activity. The nutritional intervention limited to the suckling period was sufficient to permanently affect gut microbiota. The infant formula composition impacted genera mainly belonging to *Clostridia* class (*Lachnospiraceae*, *Ruminococcaceae* and *Peptostreptococcaceae* families): *Hungatella*, *Lachnospiraceae* AC2044 group, *Peptoclostridium*, *Flavonifractor* and *Lachnoclostridium* were increased in DL and/or DL + Lf pigs compared to PL pigs. In our study, strong positive correlations (R > 0.70, p ≤ 0.01) were observed between OTUs, especially between the ones belonging to *Ruminococcaceae* and *Lachnospiraceae* families. These are the most abundant families from the phylum Firmicutes (and order *Clostridiales*) found in the mammalian gut environment: 10–20% of the total bacteria in the human colon^26^ and 18.7% in faecal samples from weaned pigs^27^. The dominant genus in the large intestine of six-month-old pigs also belongs to *Clostridiales*^28^. *Lachnospiraceae* and *Ruminococcaceae* have been associated with gut health maintenance through SCFA production^29^. An increase in *Clostridia* class (butyrate-producing bacteria) was associated with a protective microbiota and with gut function and immune homeostasis maintenance in both mice and humans^30–32^ although no change in immune parameters was observed in our study. In addition, *Clostridia* class was shown to be significantly decreased in diabetic patients^33,34^ and in rats fed a high-fat high-sucrose diet^35^ although some studies demonstrated an enhancement of *Clostridiales* order in response to an adverse diet or obesity^36–38^. However, in our study, increased abundance of these genera in DL and/or DL + Lf pigs compared to PL pigs had no consequences on glucose tolerance after three months of HE diet challenge. All three groups of animals displayed an insulin-resistance state (elevated HOMA-IR values) and an impaired glucose tolerance (IVGTT) but not diabetes, as demonstrated by fasting glycaemia within the physiological range.

The increase of *Lachnoclostridium* in DL + Lf compared to PL pigs is of particular interest since it can ferment mono- and disaccharides and produce SCFAs^39^. Some members of *Lachnoclostridium* were found to be close to *Clostridium lactatifermentans*, a strain that ferment lactate to SCFAs (mainly acetate and propionate and also traces of butyrate and isovalerate)^40^ and *Lachnoclostridium* abundance was decreased in mice fed a high-fat diet^41^. In our study, *Lachnoclostridium* was positively correlated with *Hungatella* and *Flavonifractor* (Fig. 5). *Lachnoclostridium* was positively associated with caecal isovalerate and *Hungatella* with caecal total SCFAs (R = 0.46) and acetate. SCFA production was also increased in DL + Lf compared to DL after six weeks (PND98, total SCFAs, acetate, isobutyrate, valerate and isovalerate) and three months (PND133 and 140 for faecal propionate) of HE diet. The infant formula containing dairy lipids and Lf compared to PL was therefore associated with both a long-term increased abundance of genera belonging to *Clostridia* class and an increased SCFAs concentration. These results suggest a beneficial effect on microbiota composition and metabolic activity since a decreased SCFA production has generally been associated with obesity^42^ and adverse diets^43,44^.

The infant formula composition also affected the faecal concentration of 4 metabolites: lysine, 5-aminovalerate, 2-oxoglutarate and glycerol. Lysine, 5-aminovalerate and 2-oxoglutarate were decreased in DL + Lf compared to PL (and also compared to DL for 5-aminovalerate) whereas glycerol was increased in DL and DL + Lf compared to PL. 5-aminovalerate is thought to be a metabolite of protein catabolism (such as lysine) by anaerobic bacteria^45,46^. It can further be degraded to acetate, propionate, ammonia and valerate and is a substrate of 4-aminobutyrate-2-oxoglutarate aminotransferase. Higher expression of microbial genes involved in the metabolism of essential amino acids (lysine) was observed in mice transplanted with obese twin microbiomes^42^ suggesting that such an increased microbial protein metabolism was associated with altered host health. Thus, in our study, the decrease in lysine and 5-aminovalerate in DL ± Lf would be beneficial for the host. Besides, 2-oxoglutarate, an intermediate of the Krebs cycle, was decreased in the urine of rats fed a high-fat/high-sucrose diet^47^ and negatively correlated with the faecal abundance of SCFAs in control rats^48^ suggesting once again that its decrease in DL + Lf was consistent with the increased SCFA production. The colonic level of glycerol, a trihydroxy sugar alcohol intermediate in carbohydrate and lipid metabolism, is partly dependent on *in situ* microbial synthesis and fermentation processes. Indeed, some fermentation products such as butyrate may stimulate glycerol absorption^49^ whereas several alcohols such as 1,3-propanediol inhibit it^50^. It was demonstrated that a slower glycerol metabolism resulted in higher levels of propionate^51^ which seems in agreement with the increased levels of glycerol and propionate observed in DL + Lf. Even if the interpretation of modulation of gut microbiota is not an easy task due to substantial discordance between studies owing to the population or animal model chosen, the methods used and the hypervariable region of the 16S gene examined, all the effects observed in our study suggest a beneficial effect of the addition of dairy lipids with Lf in infant formula on later gut microbiota composition (increased abundance of *Clostridiales* genera) and metabolism (increased SCFAs and decreased oxoglutarate).

Gut microbiota has been associated with several host metabolic pathways such as the stimulation of the intestinal endocrine function, mainly via SCFAs^52–54^. In our study, the three discriminating genera, *Flavonifractor*, *Lachnoclostridium* and *Hungatella*, increased in DL + Lf compared to PL, were positively correlated to plasma GLP-1 secretory response to meal stimulation, suggesting that they indeed participated to enhanced intestinal endocrine function observed in DL + Lf animals (Fig. 5). Besides, *Lachnospiraceae* AC2044 genus (more abundant in DL + Lf than in PL and DL) was positively correlated to colonic GLP-1 density, fasting plasma GLP-1 and fasting insulinemia. Plasma GLP-1 secretory response to meal stimulation was, as expected, positively associated with all SCFA concentrations measured at PND133 (including acetate, propionate and butyrate). These results highlight potential mechanisms between bacteria composition and metabolism, and host metabolism. Among SCFAs, propionate, which was constantly increased in DL + Lf, would be the most potent agonist of FFAR2 and FFAR3 for GLP-1 secretion^55^. *FFAR3* expression in both the caecum and the colon was positively correlated to the percentage of caecal GLP-1 secreting-cells amongst enteroendocrine cells suggesting mechanistic links between SCFAs, *FFAR* expression and intestinal endocrine function.

Accordingly, caecal GLP-1 concentration (basal condition) and plasma secretory response to meal stimulation (challenged condition) were significantly increased in DL + Lf group compared to PL and DL groups. This reflects an improved enteroendocrine cell function in DL + Lf animals, which may help them to better cope with the HE-diet challenge. Indeed, obesity and metabolic changes occurring with the development of type-2 diabetes have generally been associated in humans with an impaired postprandial secretion of GLP-1^56^. In addition, high-fat diet consumption has been associated with a decreased number of GLP-1 positive cells in the colon of mice, and with a decreased response of primary small intestinal L-cell cultures to nutrient stimuli, suggesting an impairment of the enteroendocrine cell function by a chronic high-fat diet^57^.

An improved intestinal endocrine function may be beneficial to glucose homeostasis by stimulating insulin secretion and preventing glucose intolerance^56^. Even if the infant formula composition had no long-term effect on global metabolism and glucose tolerance in our study, caecal GLP-1 L-cell density was positively correlated to the pancreatic endocrine tissue in accordance with the well-known trophic effect of GLP-1 on β cells^58^ (Fig. 5). Furthermore, GLP-1 secretory response to meal stimulation was negatively correlated to *INSR* expression in the liver, reinforcing the hypothesis that increased GLP-1 secretion may be an adaptive mechanism to slow down the establishment of glucose intolerance^59^. Besides, the decreased hepatic expression of neoglucogenic enzyme G6P in DL and DL + Lf liver suggests better insulin sensitivity in these groups compared to PL. Several hypotheses can be made to explain the lack of clear long-term outcomes of infant formula composition on metabolism. The objective of the HE diet provided in adulthood was to challenge the metabolic adaptation capabilities of the animals, to reveal a potential programming effect of the infant formula composition. Our model was successful, since we were able to demonstrate a programming effect on microbiota and its metabolism, as well as on the intestinal endocrine function but the challenge duration might have been too short to reveal their consequences on metabolism adaptation to the HE diet. Besides, pigs received the HE diet during puberty which could have challenged more drastically their metabolism^60^. It is however plausible that their increase in GLP-1 secreting capacity would help DL + Lf pigs to metabolically cope with the HE diet on a longer term.

To conclude, our study highlighted a programming effect of the infant formula composition, *i.e*. the fat matrix and the addition of probiotic Lf, on gut microbiota composition and metabolism. However, only the addition of Lf had a long-term promoting effect on the growth of intestinal SCFA-producing bacteria from the *Clostridiales* order (such as *Lachnoclostridium*) accompanied by an increase in SCFA production (especially propionate) and GLP-1 secreting function under a HE-diet challenge (Fig. 5). The addition of dairy lipids and Lf in infant formulas would, therefore, be safe on the long-term and may represent a clever way to better approach the long-term physiological effects of breast milk.

## Methods

### Ethical Approval

The present study was designed and conducted in compliance with the current ethical standards of the European and French guidelines (directive 2010/63/EU and decree 2013-118, respectively). The local Ethics Committee CREEA (Rennes Committee of Ethics in Animal Experimentation) approved the entire protocol (authorization #2016011111546978). Animals were observed daily throughout the experimental protocol to ensure their welfare and they received no medication or antibiotic treatment.

### Animals and study design

A total of 27 female and male Yucatan piglets (Saint-Gilles, France) were used in three replications. One animal was excluded because of medical issues. Piglets were separated from their dam at PND2 and housed in individual stainless-steel metabolic cages. They were fed one of the three experimental formulas with an automatic milk feeder as previously described^61^ until weaning, at PND28. To account for litter-to-litter variation, three piglets with a body weight (BW) close to the mean birth weight of the litter were selected from each litter and assigned to one of the three formulas. Allocation to formulas was balanced between groups for birth weight, BW at PND2 and sex. Birth weight was similar in all groups (791 ± 32 g). Formulas were rehydrated each day at 20% dry extract in water before distribution. The ration was allocated in ten meals automatically distributed during the day. BW was measured twice a week and feeding schedules were adjusted accordingly. The daily net energy offered was 1450 kJ/kg BW^0.75^. Formula intake was automatically recorded daily. At weaning, pigs were fed commercial starter diets (Cooperl Arc Atlantique) until PND56 and then a HE diet until euthanasia at PND140 (Fig. 1). The HE diet was formulated to provide 37.8% of digestible energy from lipids and 45.2% from carbohydrates -mainly sucrose-, to mimic an obesogenic environment (*vs*. 2.2% from lipids and 75.2% from carbohydrates -mainly fibers- in a standard minipig diet). Pigs had *ad libitum* access to water and food from PND28 to PND140. Solid food intake was recorded weekly (and daily one week before and two weeks after the administration of the HE diet) and BW was measured weekly. At PND130, plasma lipid profile was analysed and glucose tolerance was assessed *in vivo* by an intravenous glucose tolerance test (IVGTT). Pigs were then euthanized at PND140, after 12 weeks of HE diet, and tissues collected and weighed.

### Diets

The three infant formulas were separately manufactured by Lactalis (Retiers, France) under the same conditions, using the same raw materials except for some plant oils and dairy lipids that were different between the PL formula and the two formulas that contained dairy lipids (DL and DL + Lf). Probiotic Lf was added at the end of the process, directly in the dry powder. Each infant formula was manufactured in one batch before the start of the experiment and was used for the three replications. The formulas were adapted to meet piglet energy and protein requirements. The three formulas had the same energy, protein, lipid and carbohydrate levels. They differed by the fat origin [only plant lipids (PL) *vs*. half-half plant lipids and dairy lipids (DL) and the supplementation with Lf (DL + Lf)] (Table 1). Compared to a standard infant formula, the experimental formulas contained higher amounts of proteins and lipids and a lower amount of lactose. However, lipids:proteins and linoleic acid:α-linolenic acid (ω6:ω3 = 6) ratios were kept similar to those found in infant formulas. The formulas were made of a mixture of skim milk powder to reach a casein:whey proteins ratio of 30:70 w/w. The HE diet, used to reveal a nutritional programming, contained 20% fat and 20% sucrose (Supplementary Table 8).

### Body composition

The adiposity was evaluated with the back fat thickness before the administration of the HE diet (PND56) and at the end of the experiment (PND140). Back fat thickness was measured ultrasonically (Sonolayer SAL-32B, Toshiba, Tokyo, Japan) on both sides at the P2-position.

### Faecal sample collection

Faeces were collected at different stages of development: at PND 33, 55, 61, 77, 98 and 133 to determine the fermentative activity of the gut microbiota (Fig. 1). They were mixed with 0.5% ortho-phosphoric acid solution, centrifuged at 1700 g for 15 min at 4 °C and supernatants collected and stored at −20 °C until further analysis of SCFA content by gas chromatography^62^.

### Blood sampling and intravenous glucose tolerance test (IVGTT)

At PND125, a catheter was inserted under general anaesthesia into one external jugular vein of the pigs. Blood was collected in 9 mL heparin-coated tubes before culture of the PBMCs, or in tubes containing K2-EDTA (0.8 M, 10 µL/mL of blood) to assay plasma cytokines. The IVGTT was performed two to five days after surgery, after an overnight fast. It consisted in multiple blood samplings before (t = −15, 0 min, basal samples) and after (t = 2, 4, 6, 8, 10, 15, 20, 25, 30, 40, 50, 60 and 75 min) an intravenous glucose injection (0.3 g/kg BW). After the IVGTT, a meal test was performed. 100 g of the HE diet were given to pigs and blood samples were collected 30 min after the meal. Blood was collected in tubes containing K2-EDTA for glucose, insulin, inflammatory markers and lipid profile and in tubes containing K2-EDTA plus an anti-dipeptidylpeptidase-IV (DPP-IV, 10 µL/mL of blood) for GLP-1 (Millipore, Billerica, MA, USA). Tubes containing “Choay” heparin were used for LPS assay. After centrifugation (10 min, 2500 g, 4 °C), plasma samples for glucose, insulin, inflammatory markers and lipid assays were stored at −20 °C and the ones for GLP-1 assay at −80 °C. Glucose and insulin assays were performed on all plasma samples whereas haptoglobin and lipid profile were only performed on basal samples. GLP-1 assays were performed on basal samples and 30 minutes after the meal test.

### Post Mortem Tissue Collection

At PND140, after an overnight fast, pigs were euthanized in the experimental slaughterhouse by electrical stunning immediately followed by exsanguination. Liver, pancreas, visceral adipose tissue, caecum and colon (full and empty) were weighed. Digestive contents from caecum, colon and faeces were collected, weighed, and 200–300 mg were immediately frozen in liquid nitrogen and stored at −80 °C for microbiota analysis and metabolomics. In addition digestive contents were mixed with 1 ml of 0.5% ortho-phosphoric acid solution per g of digesta for quantification of SCFAs. Around 1 g of tissues were frozen in liquid nitrogen and stored at −80 °C for further analysis. Adjacent pieces of caecum and colon were fixed in 4% paraformaldehyde for immunohistochemistry. For determination of gene expression, liver, caecal and colon samples were kept in a RNA later solution (Thermo Fisher Scientific, Villebon-sur-Yvette, France) for 24 h at 4 °C and stored at −20 °C. One cm^3^ sample of the body of pancreas was directly frozen and stored at −80 °C for insulin extraction and another cm^3^ was fixed in 4% paraformaldehyde for immunohistochemistry. Finally, caecal and colon tissues were collected, rinsed with cold PBS and stored at −80 °C until GLP-1 extraction and assay.

### Faecal microbiota analyses

#### DNA extraction and sequencing

Extraction of total bacterial DNA from rectal contents was performed using Guanidium Thiocyanate and the mechanical bead-beating disruption method as previously described^63^. DNA concentration was determined with DeNovix spectrophotometer (Wilmington, USA). The V3-V4 region of the 16S rRNA gene was amplified for 30 amplification cycles with an annealing temperature of 65 °C by using the primers F343 (CTTTCCCTACACGACGCTCTTCCGATCTACGGRAGGCAGCAG) and R784 (GGAGTTCAGACGTGTGCTCTTCCGATCTTACCAGGGTATCTAATCCT). The amplicon lengths were about 450 bp (depending on the species). Because MiSeq sequencing enables paired 250 bp reads, the ends of each read overlap and can be stitched together to generate extremely high-quality, full-length reads covering the entire V3-V4 region. Single multiplexing was performed using a home-made 6 bp index, which was added during a second PCR with 12 cycles using the forward primer (AATGATACGGCGACCACCGAGATCTACACTCTTTCCCTACACGAC) and the modified reverse primer (CAAGCAGAAGACGGCATACGAGAT-index-GTGACTGGAGTTCAGACGTGT). The resulting PCR products were purified and loaded onto the Illumina MiSeq cartridge according to the manufacturer instructions. The quality of the run was checked internally using PhiX, and for further analysis, each paired sequence was assigned to its sample using the previously integrated index.

#### Bioinformatics and statistical analysis

The 16rRNA raw sequences were analysed using the bioinformatic pipeline FROGS (Find Rapidly OTU with Galaxy Solution)^64^. After performing the quality control depletions, 458,295 total sequences (out of the 674,981 initial sequences) with a mean of 20,831 sequences per sample were used for the analyses. Reads were classified into 909 OTUs. OTUs were assigned using the silva123 16S database. Multi-affiliation was manually checked: all sequences with a low pintail quality in the silva database were removed; a single affiliation was accepted when corresponding to more than 90% of all OTU sequences. The phylogenetic tree was constructed using Phy_tree. After multi-affiliation management, the resulting OTU table (unrarefied raw data) was used for subsequent statistical analyses. Data were filtered to keep only those that contributed to at least 0.1% of the microbial community in at least 5% of the samples. Differences in phyla, genera and OTUs, were assessed with pairwise comparisons, after aggregation at the desired taxonomic rank (phyloseq’ tax_glom function) by using EdgeR package (Bioconductor)^65^, correcting for a sex effect. Multiple testing corrections (False Discovery Rate) were used to avoid false positives (significance threshold = 0.05).

To investigate within- and between-sample diversities (α and β, respectively), all samples were rarefied to the same depth (15,000 sequences) before analysis with phyloseq R package. α diversity was studied using species richness, Chao1, Simpson index, Shannon index, and inverse Simpson index. Significant differences between formulas and their interactions with sex were assessed using ANOVA (aov function). Compositional β diversities were studied using Jaccard and Bray-Curtis distances whereas phylogenetic β diversities were studied using Unifrac and Weighted Unifrac distances. Group differences in β diversity measurements were evaluated with principal co-ordinate analysis (PCoA) and permutational multivariate analysis (PERMANOVA) of variance using distance matrices. Significance was assessed with adonis and anosim statistical tests (vegan package of R, infant formula composition × sex model) to evaluate the distances at 9999 permutations between groups.

### Metabolomics

#### Sample preparation and 1H-Nuclear Magnetic Resonance (NMR) spectroscopy

Faecal water extraction protocol was adapted from Zhao *et al*.^66^. Briefly, 500 µL of phosphate buffer (pH 7.0) prepared in D_2_O were added to 100 mg of rectal content, homogenized using a FastPrep (MP Biomedicals), centrifuged at 15,000 g for 10 min at 4 °C and supernatants were kept aside. 500 µL of phosphate buffer (pH 7.0) prepared in D_2_O were added to the pellets and processed as above. The two supernatants were pooled and centrifuged (15 min, 4 °C, 15,000 g) before being transferred into 5 mm RMN tubes. All ^1^H-NMR spectra were obtained on a Bruker Avance III HD NMR spectrometer operating at 600.13 MHz for ^1^H resonance frequency using an inverse detection 5 mm ^1^H-13C-15N-31P cryoprobe attached to a CryoPlatform (the preamplifier cooling unit). ^1^H NMR spectra were acquired at 300 K using the Carr-Purcell-Meiboom-Gill (CPMG) spin-echo pulse sequence with presaturation, with a total spin-echo delay of 240 ms to attenuate broad signals from proteins and lipoproteins for faecal samples. A total of 512 scans were collected, using a spectral width of 20 ppm and a relaxation delay of 2 s. All spectra were Fourier transformed, manually phased, baseline corrected and referenced to TMSP using Bruker TopSpin 3.5 software (Bruker, GMBH, Karlsruhe, Germany). The spectral assignment was based on matching data to reference spectra in a home-made reference database.

#### Data reduction and multivariate statistical analysis

Data were reduced using the AMIX software (version 3.9.11, Bruker, Rheinstetten, Germany) to integrate 0.01 ppm regions corresponding to the δ 9–0.5 ppm region. To account for differences in sample amount, each integrated region was normalized to the total spectral area.

SIMCA-P + software (V13, Umetrics AB, Umea, Sweden) was used to perform the multivariate analyses. Before multivariate analysis, Orthogonal Signal Correction (OSC) filtering^67^ was used to remove variation not linked to the infant formula composition. Filtered data were mean-centered and Pareto scaled. Principal Component Analysis was firstly applied to detect outliers and intrinsic clusters. Then, Partial Least Squares – Discriminant Analysis (PLS-DA) was used to assess the relationship between diet (infant formula) and metabolomic profiles. PLS-DA is a supervised method that maximizes the separation between groups. The R^2^ (percentage of explained variance) and Q^2^ (predictive capacity of the model) criteria were used to assess model performance. Models were considered to be valid and robust when R^2^ (percentage of explained variance) > 50% and Q^2^ (predictive capacity of the model) > 40%^68^. Permutation tests involving 200 iterations were also used to assess the robustness of the models. The number of latent variables and Q² value were calculated using seven-fold cross validation. Discriminant variables were determined using VIP (Variable Importance in the Projection) and Kruskal-Wallis test was applied to assess which variables were significantly different. Multiple testing corrections (False Discovery Rate) were used to avoid false positives (significance threshold = 0.05).

### Immunohistochemical analysis

Fresh caecum, colon and body of the endocrine pancreas samples were fixed in 4% paraformaldehyde for 72 h at room temperature. They were then placed at 4 °C in PBS containing 30% sucrose and embedded in the Tissue-Tek Optimum Cutting Temperature compound (Sakura Finetek Europe B. V., Zoeterwoude, The Netherlands), frozen in isopentane and sectioned (10 μm for pancreas and 7 µm for caecum and colon) using a cryostat-microtome. Caecum and colon sections (n = 6 per group) were rehydrated with PBS and incubated with 4% normal horse serum, Triton X-100 (0.5%) and sodium azide (0.1%) in PBS for 30 min at room temperature. Sections were then exposed overnight to a rabbit anti-GLP-1 antibody (1:200; Abcam, Cambridge, UK). After washing with PBS, they were incubated with an anti-rabbit antibody conjugated with Alexa Fluor 555 (1:500; Cell Signaling Technology, Leiden, The Netherland) for 2.5 h. Sections were then washed with PBS, incubated with a mouse anti-Chromogranin A antibody (1:1000; Abcam, Cambridge, UK) for 3 h, washed with PBS, incubated with an anti-mouse antibody conjugated with Alexa Fluor 488 (1:500; Cell Signaling Technology, Leiden, The Netherlands) for 2.5 h, washed again with PBS and cover-slipped with Vectashield. Sections were examined by using a fluorescence microscope (Eclipse E400, Nikon Instruments France, Champigny-Sur-Marne, France) attached to a digital camera (Digital Still DXM 1200, Nikon Instruments France). The whole section of the tissue was scanned using digital slide scanner (Nanozoomer 2.0-RS, Hamamatsu Photonics France SARL, Massy, France). Recorded files were analysed using the software NDPI (Hamamatsu Photonics) for determination of the number of enteroendocrine (chromogranin A-labelled) cells and GLP-1-secreting cells per area of mucosa. Immunohistochemical analysis of pancreas (n = 7 per group) was processed as previously described^69^ to obtain the percentage of endocrine tissue and the number and diameter of islets.

### Hormone, glucose, lipid and inflammatory marker assays

Plasma glucose, non-esterified fatty acids (NEFA), triglycerides, total cholesterol, High-density lipoprotein (HDL)-cholesterol and haptoglobin were assessed by an automated spectrophotometric method (Konelab 20i, Thermo Fisher Scientific, Illkirsh, France) using specific commercial kits (Biomérieux, Bruz, France). The intra-assay coefficient of variation was <5%.

GLP-1 content was extracted from caecum and colon by homogenisation (Polytron 3100, Kinematica, 25,000 rpm, 2 × 20 s) of 1 g of tissue in 5 ml of ethanol acid solution (1% HCl 12 M, 74% absolute ethanol, 25% H_2_O). After 24 h at 4 °C, samples were centrifuged (20 min, 2000 g, 4 °C) and supernatants diluted (1:300 and 1:250 for caecum and colon, respectively). Intestinal and plasma GLP-1 concentrations were measured using a GLP-1 active ELISA kit (Millipore). Non-responding pigs to meal-stimulation were removed from the secretory response analysis.

Insulin content was extracted from the pancreas by homogenisation in 10 mL of an ethanol acid solution (75% absolute ethanol, 23.5% ultrapure H_2_O, 1.5% HCl 12 N) (Polytron 3100, Kinematica, 25,000 rpm, 2 × 20 s). After an overnight storage at −20 °C, samples were centrifuged (30 min, 190 g, 4 °C) and supernatants stored at −20 °C. Ethanol acid solution (10 mL) was added to pellets for a second extraction, stored overnight at −20 °C and centrifuged (30 min, 190 g, 4 °C). Supernatants were collected and pooled to the ones from the first extraction, diluted in a PBS/BSA solution (1:3000) and pancreas insulin concentration and plasma insulin were measured by a radioimmunoassay (RIA) method, using iodinated porcine insulin as a tracer (INSULIN-CT, CisbioInternational, Gif sur Yvette, France). The intra- and inter-assay CV were 15 and 11% respectively for a concentration of 35 µIU/mL.

Plasma cytokines (IL-1β and TNFα) were measured using Cloud-Clone Corp kits (USA, SEA563Po and SEA133Po, respectively) according to manufacturer’s instructions.

### Gene expression in liver, caecum and colon tissues

#### Total RNA extraction, integrity and quantitative RT-PCR

Total RNA was isolated from liver, caecal and colon samples stored in RNA later solution according to the TRIzol method (Invitrogen, France). Extracted RNAs were quantified using a DeNovix spectrophotometer (Wilmington, USA). The RNA quality and integrity were confirmed using the Agilent RNA 6000 Nano kit utilizing Agilent 2100 Bioanalyzer (Agilent Technologies France, Massy, France). After a DNase treatment (Ambion), cDNA was prepared by reverse transcription of 2 μg total RNA using a High Capacity Complementary DNA Reverse Transcription Kit according to the manufacturer’s instructions (Applied Biosystems). Quantitative real-time PCR was performed on a StepOnePlus real-time PCR machine using Fast SyberGreen master mix (Applied Biosystems) for detection.

*RPL4*, *GAPDH* and *YWHAZ* were used as housekeeping genes in the liver while *TBP* and *ACTB* were used as housekeeping genes in the caecum and colon according to their stability (Supplementary Table 9). Relative expressions of the target genes were determined using the 2^−ΔΔCt^ method. Target genes in the liver were genes encoding gluconeogenic enzymes: G6Pase and PEPCK (phosphoenolpyruvate carboxykinase), and genes related to insulin action: GLUT2 (glucose transporter 2) and INSR (insulin receptor). Target genes in the caecum and the colon were *FFAR 2* and *3*.

### Insulin resistance and minimal model

Insulin resistance was evaluated using the Homeostasis Model Assessment Insulin Resistance Index (HOMA-IR = (G_0_ (mmol.L^−1^) × I_0_ (µIU.mL^−1^)/22.5) where G_0_ and I_0_ are fasting glycemia and insulinemia, respectively. Glucose tolerance was estimated through the following indexes: the incremental area under the curve (AUC) over 30 and 75 min for glucose and the rate of glucose disappearance (*K*_*G*_, slope of the function f(t) = Ln(G), in %/min). Beta-cell secretory capacity was estimated through the calculation of the AUC over 30 and 75 min for insulin, the acute insulin response (AIR, mean insulin concentration above basal values for the first 6 min) and the insulinogenic index (AUC_insulin_/AUC_glucose_) at 30 and 75 min.

The glucose and insulin responses were integrated using the minimal model described by Bergman^70^ to obtain the glucose efficiency index S_G_ and the insulin sensitivity index S_I_ (SAAM Institute Inc, Seattle, WA, USA). The S_2_ index, described to be well-correlated to the insulin sensitivity index obtained by the gold standard euglycemic hyperinsulinemic clamp^71^ was also calculated.

### Alkaline phosphatase activity in caecum and colon

Bioactive phosphatase alkaline concentration was determined in caecum and colon with a commercial kit (Sensolyte®, Anaspec, San Jose, USA) and expressed per mg of soluble protein.

### Culture and stimulation of PBMCs

Peripheral blood was diluted with an equal volume of Hank’s Balanced Saline Solution (HBSS) supplemented with 2% foetal calf serum (FCS), 100 IU/mL penicillin and 100 µg/mL streptomycine sulphate. Diluted blood (6 mL) was then layered over 2.5 mL of Ficoll-Hypaque (Histopaque-1077, Sigma, #10771) and centrifuged at 400 g for 30 min at room temperature without brake. Mononuclear cells were collected at the interface between Ficoll and plasma. HBSS-FCS was added to mononuclear cells up to a total volume of 50 mL and centrifuged 10 min at 600 g and 4 °C. Then the pellet was suspended in 10 mL of room temperature ACK lysing buffer to lyse red blood cells, if any. After 5 min, reaction was stopped by adding 10 mL of ice-cold complete RPMI medium (supplemented with 10% of FCS). PBMCs (1.25 × 10^6^ cells per well in flat-bottomed 96 well plates) were cultured for 72 h at 37 °C under an atmosphere containing 5% CO_2_. Three different conditions of culture were performed: unstimulated condition (only with RPMI complete medium), in the presence of 5 µg/ml ConA (Sigma, C5275, Saint Quentin Fallavier, France), or in the presence of 10 µg/ml LPS-EB ultrapure from *Escherichia coli* 0111:B4 strain (tlrl-3pelps, InvivoGen, San Diego, USA). Finally, supernatants were collected and frozen at −20 °C for later cytokine analysis by ELISA. Indeed, IL-10, IFNγ and TNFα were measured in culture supernatants of PBMCs using capture sandwich ELISA (R&D Systems, USA: DY693B, DY985 and DY690B, respectively). Secretion levels (pg/mL) were obtained by subtracting basal condition level to stimulated condition levels.

### Statistical analysis

Statistical analyses of microbiota and metabolomics data were described above. For all other data, statistical analyses were performed using R Core Team (2016; R: A language and environment for statistical computing. R Foundation for Statistical Computing, Vienna, Austria; http://www.R-project.org/). Normality was tested with Shapiro and Wilk test. For parameters with unequal variances between groups, Box-Cox transformations were used. Differences between groups were assessed using a two-way ANOVA (lm function) testing formula, replication, sex and interactions between diet and sex, and between diet and replication, followed by Tukey post-hoc test (Tukey HSD).

BW, energy intake, glucose and insulin responses to IVGTT, FFAR expression across sites were subjected to repeated measures ANOVA (lme function) including diet, time (age or min after glucose injection), site (caecum and colon for *FFAR* expression only), replication, sex and interactions between diet and all other factors, followed by Tukey post-hoc test (lsmeans package). Sex effect and interaction diet × sex were not significant unless otherwise mentioned.

Spearman’s r-test was used to evaluate correlations between parameters for all groups. Correlations were considered when p < 0.05 and |R| > 0.5.

Data are presented as mean values with their standard errors (SEM). Differences were considered significant at p < 0.05 and a trend at p < 0.1.

### Data Availability

The datasets generated during and/or analysed during the current study, which are not already included in this published article (and its Supplementary Information files), are available from the corresponding author on reasonable request.

## Electronic supplementary material

Supplementary Information

Supplementary Table 1 - Dataset 1
