# Supplementary material for: Addition of dairy lipids and probiotic Lactobacillus fermentum in infant formula programs gut microbiota and entero-insular axis in adult minipigs
Source: Sci Rep. 2018 Aug 3;8:11656. doi: 10.1038/s41598-018-29971-w (PMC6076243; doi:10.1038/s41598-018-29971-w)
Supplement: Supplementary file 1 — Supplementary Information [file 41598_2018_29971_MOESM1_ESM.pdf]

Addition of dairy lipids and probiotic *Lactobacillus fermentum* in infant formula programs gut microbiota and entero-insular axis in adult minipigs

Marion Lemaire[1,2], Samir Dou[3], Armelle Cahu[1], Michèle Formal[1], Laurence Le Normand[1], Véronique Romé[1], Isabelle Nogret[1], Stéphanie Ferret-Bernard[1], Moez Rhimi[4], Isabelle Cuinet[2], Cécile Canlet[5], Marie Tremblay-Franco[5], Pascale Le Ruyet[2], Charlotte Baudry[2], Philippe Gérard[4], Isabelle Le Huërou-Luron[1#], Sophie Blat[1#]*

Supplementary Table 1: Effects of dairy lipids and Lf on α and β diversities and on gut microbiota composition at phylum, genus and OTU levels

Dataset 1

Supplementary Table 2: Summary of correlation analysis of gut microbiota composition (discriminating genera or OTUs) and metabolites

**Parameter 1**	**Parameter 2**	**R**
Peptoclostridium	*Enterococcus*	0.66
Peptoclostridium	*Flavonifractor*	0.65
Peptoclostridium	*Fusobacterium*	0.58
Peptoclostridium	*Lachnoclostridium*	0.54
Lachnoclostridium	*Flavonifractor*	0.77
Lachnoclostridium	*Lachnospiraceae* AC2044 group	-0.61
Lachnoclostridium	*Hungatella*	0.57
Hungatella	*Flavonifractor*	0.55
Unknown genus (p-2534-18B5 gut group family)	Unknown genus (*Porphyromonadaceae* family)	0.84
Unknown genus (p-2534-18B5 gut group family)	*Enterococcus*	0.64
Enterococcus	*Flavonifractor*	0.63
Hungatella	Caecal acetate	0.52
Lachnoclostridium	Caecal isovalerate	0.52
Prevotella 2	Faecal propionate (at PND140)	-0.54
Prevotella 2_unknown species	Faecal butyrate (at PND133)	-0.70
Prevotella 2_unknown species	Faecal isobutyrate (at PND133)	-0.70
Prevotella 2_unknown species	Faecal valerate (at PND133)	-0.72
Unknown genus_unknown species (*Prevotellaceae* family)	Faecal valerate (at PND133)	-0.71
Faecal propionate (at PND140)	Caecal acetate	-0.50
Peptoclostridium	Faecal 5-aminovalerate (at PND140)	-0.51
Prevotella 2	Faecal 5-aminovalerate (at PND140)	0.50

Faecal 5-aminovalerate (at PND140)	Faecal glycerol (at PND140)	-0.56
Faecal glycerol (at PND140)	2-oxoglutarate	-0.77
2-oxoglutarate	Lysine	0.59

Selected correlations with $p < 0.05$ and $|R| > 0.5$. PND, postnatal day; OTU, operational taxonomic unit.

Supplementary Table 3: Entero-insular axis parameters of adult PL, DL and DL+Lf pigs

Nb/mm^2 mucosa	PL	DL	DL+Lf	p-value<br>Diet effect
**Caecum**				
Enteroendocrine cells	12.6 ± 2.2	10.3 ± 1.4	14.1 ± 2.7	0.19
GLP-1-secreting L-cells	3.47 ± 0.50	2.92 ± 0.25	3.67 ± 0.40	0.42
% GLP-1 amongst enteroendocrine cells	28.1 ± 1.1	30.1 ± 2.4	27.9 ± 2.7	0.61
**Colon**				
Enteroendocrine cells	10.3 ± 0.9	9.68 ± 1.19	10.3 ± 1.4	0.86
GLP-1-secreting L-cells	2.26 ± 0.29	2.14 ± 0.31	2.27 ± 0.48	0.94
% GLP-1 amongst enteroendocrine cells	21.9 ± 1.9	22.0 ± 1.0	21.4 ±2.3	0.96
**Pancreas**				
Pancreas weight (g)	34.6 ± 2.0	38.4 ± 1.9	37.2 ± 2.8	0.46
Pancreas weight (g/kg of body weight)	1.05 ± 0.05	1.07 ± 0.04	1.07 ± 0.08	0.97
Endocrine pancreas				
Endocrine tissue (%)	2.51 ± 0.25	2.44 ± 0.16	2.33 ± 0.26	0.68
Number of islets (per 0.5 cm^2 tissue)	227 ± 20	234 ± 19	220 ± 15	0.87
Mean islet diameter (µm)	71.4 ± 1.2	69.6 ± 1.5	69.4 ± 1.5	0.54
Insulin content* (IU/ pancreas)	510 ± 59	579 ± 70	509 ± 42	0.65

Formulas contained as lipids either: only plant lipids (PL, n=6-9), a half-half mixture of plant and dairy lipids (DL, n=6-8) or a half-half mixture of plant and dairy lipids supplemented with Lf (DL+Lf, n=6-9).

*Insulin content per pancreas was higher in males than in females ($p=0.05$). Data are expressed as the mean ± SEM. GLP-1, Glucagon-like peptide-1.

Supplementary Table 4: Significant correlations between entero-insular axis parameters

**Parameter 1**	**Parameter 2**	**R**
Caecal GLP-1-secreting L-cells	Caecal GLP-1 (g/tissue)	0.52
Caecal GLP-1-secreting L-cells	Plasma GLP-1 secretory response to meal stimulation	0.62
Caecal GLP-1-secreting L-cells	Endocrine pancreas (% endocrine tissue)	0.74
Caecal GLP-1-secreting L-cells / enteroendocrine cells	*FFAR3* caecum	0.56
Caecal GLP-1-secreting L-cells / enteroendocrine cells	*FFAR3* colon	0.59
Colon GLP-1-secreting L-cells	Fasting plasma GLP-1	0.70
Colon GLP-1-secreting L-cells	Colon GLP-1 (g/tissue)	0.51
Colon GLP-1-secreting L-cells / enteroendocrine cells	*FFAR3* colon	0.53
FFAR2 caecum	*FFAR2* colon	0.56
FFAR2 colon	*FFAR3* colon	0.55
Liver *INSR* expression	Plasma GLP-1 secretory response to meal stimulation	-0.66

Selected correlations with $p < 0.05$ and $|R| > 0.5$. GLP-1, Glucagon-like peptide-1; *FFAR*, Free fatty acid receptor; *INSR*, Insulin receptor.

Supplementary Table 5: Correlations between gut microbiota (composition and metabolites) and entero-insular axis parameters

Entero-insular axis	Gut microbiota	R
Caecal GLP-1-secreting L-cells	Total faecal SCFAs after six weeks of HE diet	0.58
Caecal GLP-1-secreting L-cells	Faecal acetate after six weeks of HE diet	0.50
Caecal GLP-1-secreting L-cells	Faecal isobutyrate after six weeks of HE diet	0.55
Caecal GLP-1 (g/tissue)	Faecal propionate after six weeks of HE diet	0.52
Caecal GLP-1 (g/tissue)	Colon butyrate	0.56
Caecal GLP-1 (g/tissue)	Faecal butyrate after three weeks of HE diet	0.51
Plasma GLP-1 secretory response to meal stimulation	*Flavonifractor*	0.62
Plasma GLP-1 secretory response to meal stimulation	*Hungatella*	0.74
Plasma GLP-1 secretory response to meal stimulation	*Lachnoclostridium*	0.75
Plasma GLP-1 secretory response to meal stimulation	Total faecal SCFAs after six weeks of HE diet	0.53
Plasma GLP-1 secretory response to meal stimulation	Total faecal SCFAs at PND133	0.53
Plasma GLP-1 secretory response to meal stimulation	Faecal acetate after six weeks of HE diet	0.57
Plasma GLP-1 secretory response to meal stimulation	Faecal acetate at PND133	0.59
Plasma GLP-1 secretory response to meal stimulation	Faecal propionate at PND133	0.59
Plasma GLP-1 secretory response to meal stimulation	Colon butyrate	0.52
Plasma GLP-1 secretory response to meal stimulation	Faecal butyrate after six weeks of HE diet	0.59

Plasma GLP-1 secretory response to meal stimulation	Faecal butyrate at PND133	0.59
Plasma GLP-1 secretory response to meal stimulation	Faecal isobutyrate at PND133	0.55
Plasma GLP-1 secretory response to meal stimulation	Faecal isovalerate at PND133	0.61
Plasma GLP-1 secretory response to meal stimulation	Faecal valerate at PND133	0.57
Colon GLP-1-secreting L-cells	*Lachnospiraceae* AC2044 group	0.65
Fasting GLP-1	*Lachnospiraceae* AC2044 group	0.54
Fasting insulin	*Lachnospiraceae* AC2044 group	0.53
Colon GLP-1-secreting L-cells / enteroendocrine cells	*Lachnoclostridium*	-0.65
Liver *INSR* expression	*Flavonifractor*	-0.52

Selected correlations with $p < 0.05$ and $|R| > 0.5$. SCFAs, Short-chain fatty acids, GLP-1, Glucagon-like peptide-1; PND, postnatal day; *INSR*, Insulin receptor; HE, high-energy.

Supplementary Table 6: Body composition throughout the experiment of PL, DL and DL+Lf pigs

	PL (n=9)	DL (n=8)	DL+Lf (n=9)	p-value Diet effect
**Weight gain (g/d)**				
PND0-28	43.7 ± 4.0 [a]	65.3 ± 7.3 [b]	60.4 ± 5.9 [a,b]	**0.02**
PND28-42	94.1 ± 20.5	98.7 ± 27.6	104.1 ± 16.5	0.88
PND42-56	210 ± 13	235 ± 26	218 ± 16	0.63
PND56-140*	328 ± 11	350 ± 11	341 ± 11	0.33
**Back fat (mm)**				
PND56	6.99 ± 0.60	8.06 ± 0.69	7.11 ± 0.52	0.42
PND140	24.4 ± 1.1	25.4 ± 1.1	23.4 ± 0.8	0.43
**Relative organ weight (PND140) (g/kg of body weight)**				
Visceral adipose tissue	34.3 ± 1.7	36.5 ± 1.5	35.2 ± 1.8	0.67
Liver*	17.6 ± 0.7	17.4 ± 1.1	17.3 ± 1.1	0.84

Data are expressed as the mean ± SEM.

Formulas contained as lipids either: only plant lipids (PL), a half-half mixture of plant and dairy lipids (DL) or a half-half mixture of plant and dairy lipids supplemented with Lf (DL+Lf).

PND, postnatal day. [a,b]Labelled means in a row without a common letter differ significantly ($p < 0.05$).

* A sex dimorphism was observed, values being significantly higher in males than females.

Supplementary Table 7: Inflammatory markers of PL, DL and DL+Lf pigs

				p-value
**Plasma inflammatory markers**	**PL**	**DL**	**DL+Lf**	**Diet effet**
Haptoglobin* (*mg/ml*)	2.63 ± 0.37	2.80 ± 0.35	3.39 ± 0.34	0.57
IL-1β* (*pg/ml*)	112 ± 13 [a]	139 ± 17 [a,b]	201 ± 35 [b]	**0.04**
TNFα (*pg/ml*)	34.6 ± 5.4	31.6 ± 1.9	35.9 ± 3.0	0.68
**Stimulated PBMC cytokine secretion (pg/ml)**				
ConA-induced IFNγ	150 ± 65	31.0 ± 10.4	126 ± 73	0.94
LPS-induced IFNγ	103 ± 100	12.3 ± 4.8	31.6 ± 10.7	0.59
ConA-induced TNFα*	1333 ± 360	759 ± 200	1354 ± 499	0.18
LPS-induced TNFα*	661 ± 277	334 ± 148	864 ± 384	0.62
ConA-induced IL-10	3345 ± 470	3213 ± 564	3063 ± 273	0.91
LPS-induced IL-10	732 ± 188	667 ± 217	1020 ± 382	0.73
**Intestinal alkaline phosphatase (mg/g protein)**				
Caecum	0.08 ± 0.01	0.06 ± 0.01	0.06 ± 0.01	0.17
Colon*	0.09 ± 0.01	0.09 ± 0.01	0.09 ± 0.01	0.87

Data are expressed as the mean ± SEM.

Formulas contained as lipids either: only plant lipids (PL, n=7-9), a half-half mixture of plant and dairy lipids (DL, n=6-8) or a half-half mixture of plant and dairy lipids supplemented with Lf (DL+Lf, n=7-9). IL-1β, Interleukin 1 beta; LPS, lipopolysaccharide; TNFα, Tumor Necrosis Factor alpha; ConA, Concanavalin A; PBMC, Peripheral Blood Mononuclear Cell. [a,b]Labelled means in a row without a common letter differ ($p<0.05$). *A sex effect was observed for haptoglobin ($p=0.04$) with higher concentrations for females than males. A diet × sex significant interaction ($p<0.01$) and sex effect ($p=0.02$) were observed for LPS-induced PBMC cell TNFα

secretion whereas only a diet × sex significant interaction ($p=0.04$) was observed for ConA-induced PBMC cell TNFα secretion.

Supplementary Table 8: Composition of the HE diet

	HE diet
**Ingredients ($g.100g^{-1}$)**	
Soybean meal	22
Saccharose	20
Wheat	12.4
Barley	12.4
Lard	10
Sunflower oil	10
Sunflower meal	10
Calcium carbonate	1.3
Bicalcium phosphate	0.6
Sodium chloride	0.6
Trace element and vitamin mix[1]	0.7
Digestible energy ($MJ.Kg^{-1}$)	17.33
Nutritional value (% digestible energy)	
Proteins	17
Lipids	37.8
Carbohydrates	45.2

The HE diet (high-energy diet) provided more saturated fat (lard and sunflower oil), more rapidly absorbed carbohydrates (saccharose) and less crude fibres than a standard diet.

Supplementary Table 9: Specific primers of porcine liver and intestinal genes

Gene	Forward (5' – 3')	Reverse (5' – 3')
Reference genes		
RPL4	CAAGAGTAACTACAACCTTC	GAACTCTACGATGAATCTTC
GAPDH	CATCCATGACAACTTCGGCA	GCATGGACTGTGGTCATGAGTC
YWHAZ	ATGCAACCAACACATCCTATC	GCATTATTAGCGTGCTGTCTT
TBP	AACAGTTCAGTAGTTATGAGCCAGA	AGATGTTCTCAAACGCTTCG
ACTB	TGTACACACCTCATGCCAGC	TCGGAGAGACTCCGTCTCAG
Target genes		
INSR	TTCTTCGAACCCCGAGTACCT	CGATGTCCCTGGCGTTTC
G6Pase	CGGCTTTCGGTGCTTGAA	CTGCACAGTCCAGAATCCCA
PEPCK	TCGAGAAAGCCTTCAATGCC	GCGTGCGACCCTTCATG
GLUT-2	TTGTTAGTCAGATCATAGGCCTCG	ATAGCTCATGATTGCCCAGGA
FFAR2	GGCTTTCCCCGTGCAGTAC	TGAACACGACGGTGCAGTGA
FFAR3	CGAGTGGAGACCTTACGTGTTG	CTTGGAACCCCGAGGATGA

RPL4, Ribosomal Protein L4; *GAPDH*, Glyceraldehyde-3-Phosphate Dehydrogenase; *YWHAZ*, Tyrosine 3-Monooxygenase/Tryptophan 5-Monooxygenase Activation Protein Zeta; *TBP*, TATA-Box Binding Protein; *ACTB*, actin beta; *INSR*, Insulin Receptor; *G6Pase*, Glucose-6-Phosphatase; *PEPCK*, PhosphoEnolPyruvate CarboxyKinase; *GLUT2*, Glucose transporter 2; *FFAR2* and *3*, Free Fatty Acid Receptor 2 and 3.

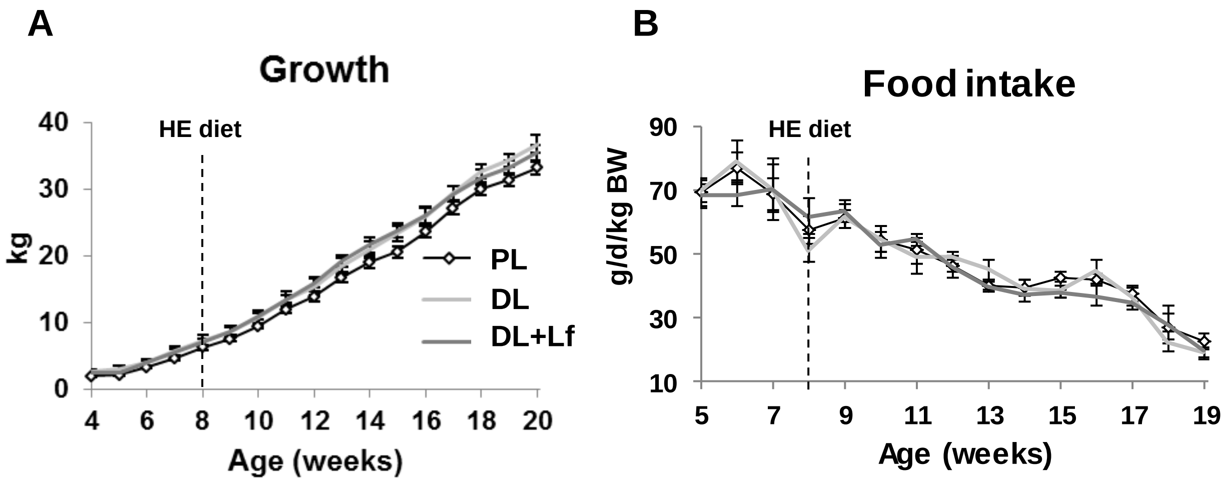

Diet p=0.19; Age p<0.01

Diet p=0.82; Age p<0.01

Supplementary Figure 1: Post-weaning growth (A) and food intake (B) of PL, DL and DL+Lf pigs

Data are shown as the mean ± SEM.

Formulas contained as lipids either: only plant lipids (PL), a half-half mixture of plant and dairy lipids (DL) or a half-half mixture of plant and dairy lipids supplemented with Lf (DL+Lf). Transition to a high-energy (HE) diet at eight weeks of age.

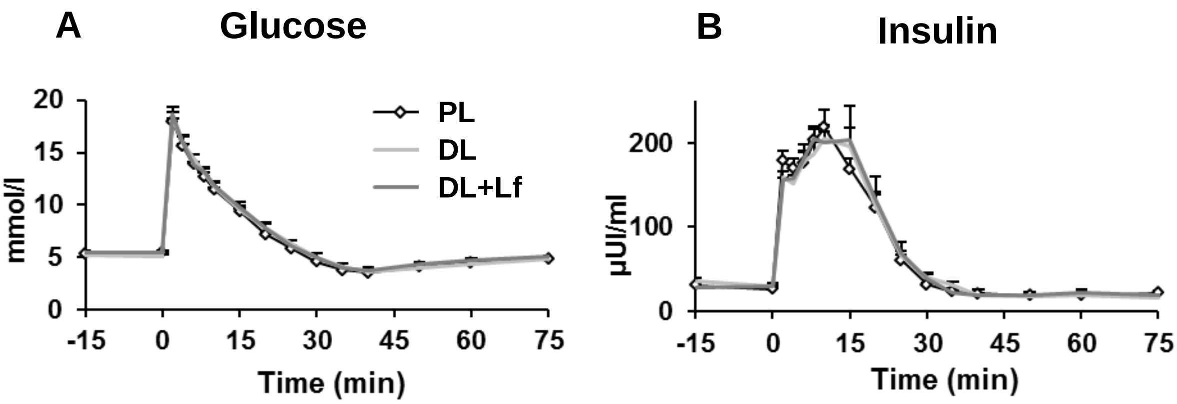

Diet p=0.85; Time p<0.01

Diet p=0.98; Time p<0.01; Sex p=0.05

Supplementary Figure 2: Plasma concentrations of (A) glucose and (B) insulin following an intravenous injection of glucose (0.3 g/ kg body weight, glucose injection at T = 0 min, right after blood sampling) of adult PL, DL and DL+Lf pigs

Formulas contained as lipids either: only plant lipids (PL, n=9), a half-half mixture of plant and dairy lipids (DL, n=8) or a half-half mixture of plant and dairy lipids supplemented with Lf (DL+Lf, n=7). Data are shown as the mean ± SEM.
